# Identification of potential ferroptosis-associated biomarkers in rheumatoid arthritis

**DOI:** 10.3389/fimmu.2023.1197275

**Published:** 2023-07-10

**Authors:** Xu He, Juqi Zhang, Mingli Gong, Yanlun Gu, Bingqi Dong, Xiaocong Pang, Chenglong Zhang, Yimin Cui

**Affiliations:** ^1^ Department of Pharmacy, Peking University First Hospital, Beijing, China; ^2^ Institute of Clinical Pharmacology, Peking University First Hospital, Beijing, China; ^3^ Department of Pharmacy Administration and Clinical Pharmacy, School of Pharmaceutical Sciences, Peking University, Beijing, China; ^4^ Department of Pharmacy, Xu Zhou Medical University, Xuzhou, China; ^5^ Department of General Surgery, Peking University First Hospital, Beijing, China; ^6^ Beijing Synchrotron Radiation Facility, Institute of High Energy Physics, Chinese Academy of Sciences, Beijing, China

**Keywords:** rheumatoid arthritis, ferroptosis, GRN, ENO1, PTGS2

## Abstract

**Background:**

Rheumatoid arthritis (RA) is a chronic autoimmune disorder characterized by inflammation and gradual joint degeneration, resulting in function disability. Recently, ferroptosis, a novel form of regulated cell death that involves iron-dependent lipid peroxidation, has been implicated in the pathogenesis of RA. However, the underlying molecular mechanisms and key genes involved in ferroptosis in RA remain largely unknown.

**Methods:**

The GSE134420 and GSE77298 datasets were downloaded and DEGs were identified using R software. The DEGs were then mapped to the dataset of 619 ferroptosis-related genes obtained from the GeneCards database. Gene Ontology (GO) and Kyoto Encyclopedia of Genes and Genomes (KEGG) pathway analyses were conducted to investigate the possible biological functions. Protein-protein interaction (PPI) networks were constructed to identify the hub genes. The relationship between hub genes and immune infiltration was estimated using the CIBERSORT algorithms. Gene Set Enrichment Analysis (GSEA) was used to explore the underlying signaling pathways of hub genes. Genome-wide association studies (GWAS) analysis was performed to confirm the pathogenic regions of the hub genes. RcisTarget and Gene-motif ranking databases were used to identify transcription factors (TFs) associated with the hub genes. The miRcode databases were utilized to construct the microRNA (miRNA)-messenger RNA (mRNA) network. Single-cell analysis was utilized to cluster cells and display the expression of hub genes in cell clusters. Finally, the expression and potential mechanism of hub genes were investigated in human and experimental samples.

**Results:**

Three hub genes PTGS2, ENO1, and GRN highly associated with ferroptosis were identified. Four pathogenic genes HLA-B, MIF, PSTPIP, TLR1 were identified that were significantly and positively correlated with the expression levels of hub genes. The results of the GSEA showed that the hub genes were significantly enriched in pathways related to immunity, lysosome, phagocytosis and infection. ENO1 and PTGS2 were enriched in the TF-binding motif of cisbp_M5493. The hub genes were validated in experimental and patient samples and highly level of ENO1 expression was found to inhibit ACO1, which reduces ferroptosis in proliferating fibroblast-like synoviocytes (FLS).

**Conclusion:**

PTGS2, ENO1 and GRN were identified and validated as potential ferroptosis-related biomarkers. Our work first revealed that ENO1 is highly expressed in RA synovium and that ferroptosis may be regulated by the ENO1-ACO1 axis, advancing the understanding of the underlying ferroptosis-related mechanisms of synovial proliferation and providing potential diagnostic and therapeutic targets for RA.

## 1 Introduction

Rheumatoid arthritis (RA) is a continuously progressive autoimmune disease with significant clinical heterogeneity. It manifests as chronic synovial inflammation, pannus formation, progressive bone erosion, and systemic inflammation that can damage multiple organ systems, including the heart, vasculature, kidneys, lungs, and nervous system (1–3). A complex interplay of various factors, including genetic, environmental factors, epigenetic, immune system dysfunction, metabolic, and microbial factors has been implicated in the pathogenesis of RA (3, 4). Although there have been significant advances in the treatment of RA over the past few decades, non-steroidal anti-inflammatory drugs (NSAIDs), disease-modifying anti-rheumatic drugs (DMARDs), corticosteroids, and biologics have shown variable efficacy in some patients (5).

The progression of RA is thought to span several decades, starting with the production of autoantibodies against post-translationally modified proteins or intracellular contents (3). This is followed by a phase of asymptomatic autoimmunity, where the immune system undergoes progressive remodeling and erosion of tissue tolerance (3). As a result, joint inflammation occurs, allowing innate and adaptive immune cells to infiltrate the synovial tissue (3). Finally, there is an irreversible phase occurs in which the transformation of synovial stromal cells into auto-aggressive effector cells transforms the acute synovitis into chronic destructive synovitis (3). Although the etiology of RA is not fully understood, it is widely accepted that oxidative stress, inflammation, and cell death play a critical role in RA development. One trigger of autoimmunity is the exposure or release of intracellular contents that activate the immune system as a result of disturbances in dead cell clearance or aberrant initiation of cell death such as necroptosis, autophagy, and pyroptosis (6). Ferroptosis is a newly discovered form of cell death characterized by iron-dependent lipid peroxidation and mitochondrial morphological changes, has recently attracted widespread scientific attention (7–11). The cellular machinery that executes ferroptosis integrates a variety of pro- or anti-survival signals from subcellular organelles and ‘chooses’ whether to initiate the lethal process (12). Ferroptosis has been implicated in the development of various pathologies, including cancer, ischemic tissue injury, infectious diseases, and neurodegeneration and has been under investigation as a potential drug target in oncology. Despite this active research into the role of ferroptosis in disease pathogenesis and drug development, its relationship with inflammatory arthritis remains in its infancy (13).

Recently, the role of ferroptosis in the pathogenesis of inflammatory arthritis has gradually been under the spotlight. Ferroptosis-modulating effects have been shown for some antirheumatic drugs, including sulfasalazine and auranofin, suggesting that the ferroptosis-modulating mechanisms in pathogenesis of inflammatory arthritis and therapeutic strategies targeting ferroptosis merit further investigation. Increased concentrations of iron, ROS and lipid peroxidation in synovial fluid and synovium in RA patients with high disease activity compared to those with moderate activity (8, 11, 14). Zhou et al. (10) observed significantly increased levels of ACSL4 and NOX4 in cartilage from RA patients and adjuvant arthritis rat model, while GPX4 was decreased, suggesting a link between increased chondrocyte ferroptosis and RA progression. In contrast to other studies, Ling et al. (14) reported opposite results, showing decreased expression of ACSL4 and increased expression of GPX4, SLC7A11 and FTH1, indicating decreased ferroptosis in RA-synovium and FLS. The existing controversies regarding the role of ferroptosis in synovial tissue of RA patients highlight the need for further research to fully understand the underlying mechanisms of this disease.

**Figure 1:**
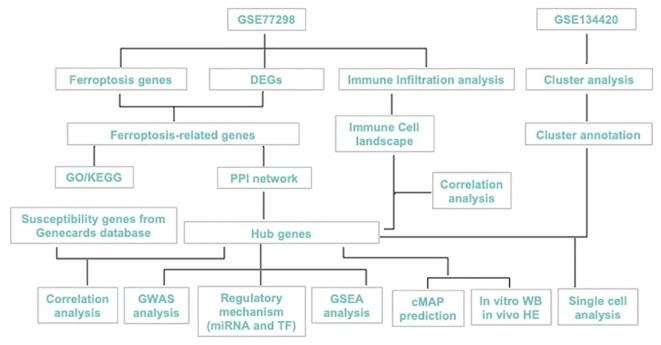
Study workflow.

Here, we aimed to explore the ferroptosis-biomarkers that provide new theoretical opportunities and therapeutic targets for developing novel drugs. PTSG2, ENO1, GRN were screened and validated, and their expression characteristics and possible regulatory mechanisms in RA were discussed using bioinformatics analysis and *in vitro* as shown in **Figure 1**, with the expectation of providing a new approach for RA diagnosis and treatment.

## 2 Materials and methods

### 2.1 Data derivation

A single cell dataset of GSE134420 was obtained from the Gene Expression Omnibus (GEO) public database for bioinformatic analysis., A total of 23 transcriptomic data sets in GSE77298, including 7 control samples and 16 RA samples, were downloaded using GPL570 platform. Differential genes (DEGs) between controls and RA were identified using R (version 3.5.1), and DEGs that were P < 0.05 and |logFC| >1 was selected for subsequent analysis. A dataset of 619 ferroptosis-related genes was retrieved from the GeneCards database (https://www.genecards.org/).

### 2.2 GO and KEGG pathway enrichment

The use of the Metascape database (www.metascape.org ) for annotation and visualization, as well as the GO analysis and KEGG pathway analysis for differentially expressed genes, is a common approach to understand the biological functions associated with disease progression. Setting a minimum overlap of 3 and a p-value threshold of 0.01 is a reasonable criterion for determining statistical significance.

### 2.3 PPI network

The PPI network of mapped genes was constructed by using the Retrieval of Interacting Genes (STRING) database (http://string-db.org/). Setting a threshold interaction score of 0.4 is a reasonable criterion for determining the strength of the interactions between genes. with a threshold interaction score of 0.4, which was visualized by the Cytoscape software. To understand the complex interactions between genes and identify key nodes in the network, Cytoscape software (version 3.7.2) was used to visualize the PPI network.

### 2.4 Evaluation of tissue-infiltrating immune cells

CIBERSORT algorithm is widely utilized to infer the relative degree of the 22 immune infiltrating cells in the microenvironment. The method is based on the principle of support vector regression (SVM), and the expression matrix of immune cell subtypes is analyzed by deconvolution. In this study, patient data was analyzed using the CIBERSORT algorithm, and the result was then used to perform Spearman correlation analysis of gene expression as well as immune cell content. By correlating gene expression with immune cell content, this analysis can provide insights into the role of different immune cell types in disease development and progression.

### 2.5 GWAS analysis

The Gene Atlas database(http://geneatlas.roslin.ed.ac.uk/) is a comprehensive resource that provides annotations against genes and proteins using the UK Biobank cohort. The database is a valuable tool for understanding the genetic basis of complex diseases and traits. The associations recorded in the Gene Atlas database have been calculated using data from 452,264 individuals from the UK Biobank. This large dataset allows for the identification of genetic variants that are associated with specific traits or diseases, providing insights into the underlying biological mechanisms.

### 2.6 GSEA analysis

GSEA analysis provides insight into the biological processes that are dysregulated in a particular phenotype or disease state by ranking genes based on their differential expression using a set of pre-defined genes, such as a pathway or gene ontology term. The method then tests whether the predefined set of genes is enriched at the bottom or top of this ranking table, indicating whether the pathway or gene ontology term is associated with the phenotype being studied. Here, we use GSEA to compare the differential signaling pathways in both the high- and low-expression group in order to investigate the underlying mechanism of key genes in both groups. Number of substitutions were 1000 and the substitution was phenotype, which means that phenotype labels were randomly permuted during the analysis.

### 2.7 Analysis of regulatory networks of hub genes

Here, we use the RcisTarget in R to predict TFs. RcisTarget calculates the AUC for each pair of motif-motif. This is the first stage to estimate the over-expression of each motif. This is done based on the calculation of recovery curve according to the order of the motifs. The normalized enrichment score (NES) is computed from AUC distributions The gene-motif ranking database used by RcisTarget is rcistarget. hg19. motifdb. cisbpont.500bp.

### 2.8 Single cell analysis

Data was primarily processed by the Seurat package, and position relationships among clusters were determined using the tSNE algorithm. The clusters were annotated by Celldex package, and some cells with relationship to RA progression were separately annotated. Finally, marker genes were extracted from single cell expression profiles with the Find All Markers logfc. threshold parameter set at 1. For each cell subtype, gene was selected to be a specific marker with |avg log_2_FC| > 1 and p val adj< 0.05.

### 2.9 Prediction of connectivity map (CMap)

The CMap is an interventional gene expression profiling database s that will be primarily used for the discovery of functional associations between small molecules, gene and disease condition. This study predicts disease-targeted therapeutics through differentially expressed genes in disease.

### 2.10 Model construction, HE staining and cell culture

Primary RA synovial fibroblasts were isolated from the synovium of male Sprague-Dawley rats weighing 160-180 g purchased from the Beijing Vital River Laboratory Animal Technology Co., Ltd. This study was approved by the Animal Ethics Committee of Peking University First Hospital. The rat model of adjuvant-induced arthritis (AIA) was established according to a previously published protocol (15). The knees of rat models were embedded in paraffin, sectioned at 5-μm thickness sections, and then stained with hematoxylin and eosin.

The methods of primary synovial fibroblast extraction and culture were based on a previous publication (16). In order to obtain pure synovial fibroblasts, cells used herein were at passage 3-5. Synovial fibroblasts were cultured in DMEM high-glucose medium (Gibico, America) containing 10% fetal bovine serum (Pan, Germany) and 1% penicillin-streptomycin (Beyotime, China) at 37 °CC in a 5% CO2 incubator.

### 2.11 Human sample, immunohistochemistry and cell culture

Synovial specimens were isolated from 3 RA patients who satisfied the diagnostic criteria of 2010 ACR and underwent total knee replacement surgery, and3 OA (osteoarthritis) patients who fulfilled the diagnostic criteria of 2010 ACR/EULAR and were undergoing surgery at the Peking University First Hospital. This study was approved by the Medical Ethics Committee of Peking University First Hospital. Detailed information on the clinical details is given in **Table S1**.

Paraffin-embedded samples were sliced into 8 μm sections. Antigen retrieval was performed using the target retrieval solution Tris-EDTA buffer with a microwave oven for 10-15 min. Tissues were immersed in 3% H_2_O_2_ for 30 min. 3% BSA was added to evenly cover the tissues for 30 min. Sections were immersed with antibodies against PTGS2(1:200, K001561P, Solarbio), ENO1(1:200, ab227978, Abcam), GRN (1:200, DF7997, Affinity Biosciences) overnight at 4°C.

Human primary FLSs were isolated from patients’ synovial tissues, minced and digested in type I collagenase (2 mg/ml, Solarbio, China) and 0.25%Trypsin-EDTA Solution (Solarbio, China) for 4 h. The resulting cell suspension was centrifuged and the cells were resuspended in medium. After 24 h of incubation, PBS washes removed nonadherent cells.

### 2.12 Construction and transfection of small interfering RNA (siRNA) and western blot

According to the GenBank database, the siRNA sequence(**Table** S2) of rat and human ENO1 gene designed and obtained from JTSBIO Co,Ltd (Wuhan, China). ENO1-siRNA and negative control (NC) vectors were transfected into primary cultured FLS cells of rat and human.

The knees of rat models were embedded in paraffin, sectioned at a thickness of 5-μm, and then stained with hematoxylin and eosin. Cells were lysed using RIPA buffer (Beyotime, China). Protein was electrophoresed with SDS-PAGE gel and transferred to PVDF membrane, blocked using QuickBlock™ Primary Antibody Dilution Buffer (Beyotime, China), and then incubated with the ENO1 antibodies (ab227978, Abcam), GRN antibodies (DF7997, Affinity Biosciences), PTGS2 antibodies (K001561P, Solarbio) and ACSL4 (ab155282, Abcam) at 4°C overnight. Membrane was subsequently incubated with HRP-conjugated secondary antibodies. BeyoECL Moon (Beyotime, China) was used to visualize the protein bands. Detection was performed using an automated chemiluminescence image analysis system (SyngeneG Box chemi xx6, British) and the quantification of the bands was performed using Image J software.

### 2.13 Cell-Counting-Kit-8 (CCK8), Cellular ferrous ion detection fluorescent probes and Lipid ROS assay

Cells were cultured in 96-well plates in 5% CO2 incubator. The medium containing the drug was replaced and add 10 microliters of CCK-8 solution (C0038, Beyotime) to each well. A blank control can be used with the corresponding amount of medium and CCK-8 added but no cells. The cells were then incubated in the cell incubator for 1.5 hours. Absorbance was measured at 450nm through Multifunctional Enzyme Labeler (SpectraMax i3).

Cells were inoculated in fluorescent culture dishes and incubated overnight in 5% CO2 incubator. The medium containing the drug was replaced, and FerroOrange working liquid (F374- DOJINDO) with a concentration of 1 μmol/l was added and incubated at 37°C in 5% CO2 incubator for 30 minutes. Fluorescent was observed under a fluorescence microscope.

To detect the level of reactive oxygen species (ROS), the medium was removed and DCFH-DA (10μmol/l, S0033S, Beyotime) was added and incubated in the incubator at 37°C for 20 minutes. The cells were washed to fully remove the excess DCFH-DA. ROS level was detected through flow cytometry (BECKMAN COULTER- CytoFLEX SRT).

## 3 Statistical analysis

Statistical analyses were performed using R language (version 4.0) and GraphPad Prism 8, presented data as mean ± standard error, and calculated statistical significance by the t-test. All statistical tests were two-sided and p<0.05 was considered statistically significant.

## 4 Results

### 4.1 Screening of DEGs

**Figure 2:**
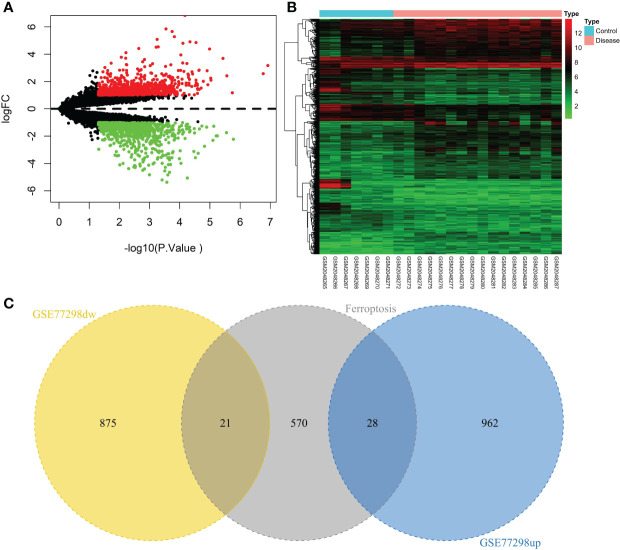
Screening of the ferroptosis-related DEGs in RA. **(A)** Volcano plot of DEGs (p < 0.05 and |log2FC| > 1). **(B)** Heatmap of DEGs. **(C)** Venn diagram of the up-regulated DEGs, the down-regulated DEGs, and the ferroptosis gene set.

A total of 23 patients, including 7 in the control group and 16 in the disease group, were downloaded from the NCBI GEO public database for the GSE77298 dataset. The limma package was used to count the differential genes between both sample groups with the differential gene screening conditions of P.value<0.05 and |logFC|>1. In total, 1886 differential genes (990 up- and 896 down-regulated genes) were finally screened (**Figure 2**). We mapped the differential genes to the ferroptosis gene set and obtained 49 overlapping genes, including 28 up- and 21 down-regulated genes.

### 4.2 Functional enrichment analysis and identifiation of hub genes

**Figure 3:**
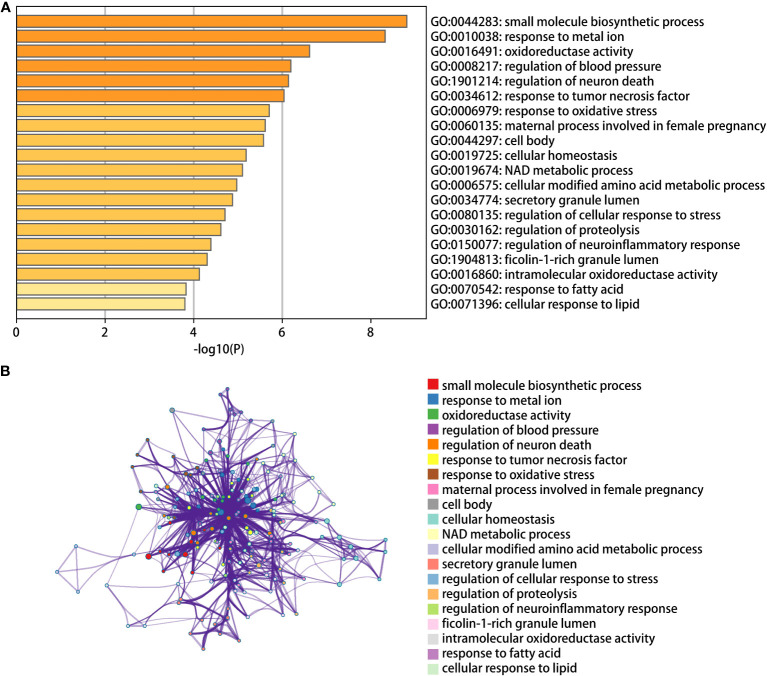
GO and KEGG pathway enrichment. **(A)** The bar chart of 20 biological pathways (p-value <0.01). **(B)** Networks enrichment terms of the 49 ferroptosis-regulated DEGs.

**Figure 4:**
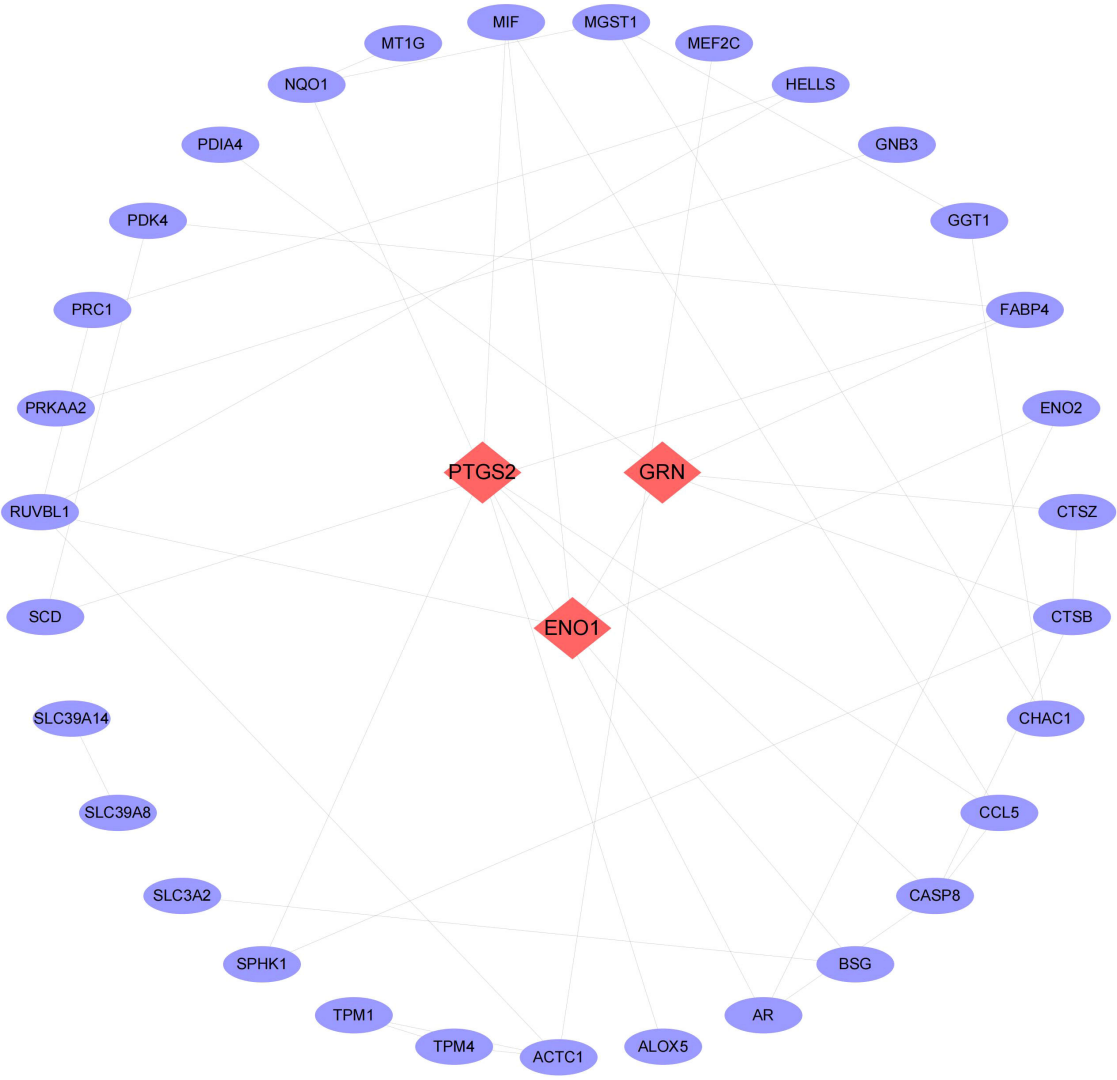
Network of PPI. The top 3 degree-value genes are shown in red rhombus as key genes.

We then conducted pathway analysis of the 49 intersecting genes, and found that these genes were primally involved in the pathways of small molecule biosynthetic process, metal ion response, and oxidoreductase activity (**Figure 3**). Protein interaction relationship pairs related to the 49 intersecting genes were retrieved from STRING database and visualized using Cytoscape, and the top 3 genes of degree were selected as key genes, which were PTGS2, ENO1, GRN (**Figure 4**).

### 4.3 Estimating of the relationship between hub genes and immune infiltration

**Figure 5:**
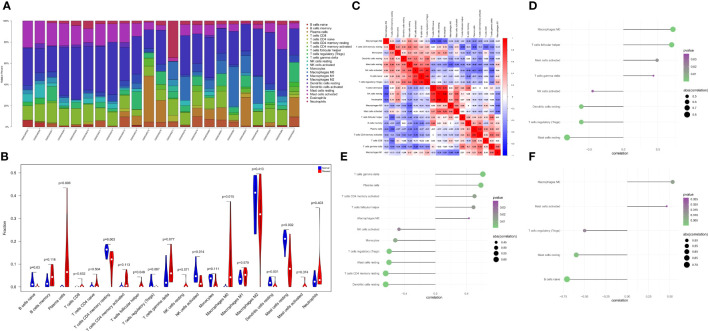
The landscape of immune infiltration between the normal and RA group. **(A)** Cumulative histogram showing the relative percentages of 22 different immune cell types. **(B)** Violin plot shows the comparisons between immune cells in normal controls and RA patients. **(C)** The heatmap shows the correlation in the infiltration of 22 immune cell type proportions. **(D-F)** Correlation between ENO1, GRN, and PTGS2 with immune infiltrating cells.

The immune microenvironment plays a crucial role in the pathogenesis of RA and influences its diagnosis, prognosis, and clinical response. By analyzing the relationship between PTGS2, ENO1, GRN and immune infiltration in the disease dataset, the mechanism by which these hub genes influence the progression of RA were explored. The level of immune cell in each sample was shown in **Figure 5A**. There were several pairs of significant correlation between the levels of immune infiltration (**Figure 5B**). And the levels of naive B cells, resting CD4 memory T cells, and activated NK cells were significantly lower in the disease group samples compared to normal patients (**Figure 5C**). We further explored the association between key genes and immune cells and found that several key genes had strong correlation with immune cells, and ENO1 had strong correlation with M0 macrophages, T follicular helper cells, and activated mast cells, and with resting mast cells, T regulatory cells (Tregs), resting dendritic cells, etc. (**Figure 5D**); GRN had a positive correlation with T gamma delta cells, plasma cells, activated CD4 memory T cells, etc., and negatively correlated with resting dendritic cells, resting CD4 memory T cells, and resting mast cells (**Figure 5E**); PTGS2 had a positive correlation with M0 macrophages and activated mast cells, and showed a negative correlation with naive B cells, resting mast cells, and Tregs (**Figure 5F**).

### 4.4 Analysis of the relationship between hub genes and the disease genes

**Figure 6:**
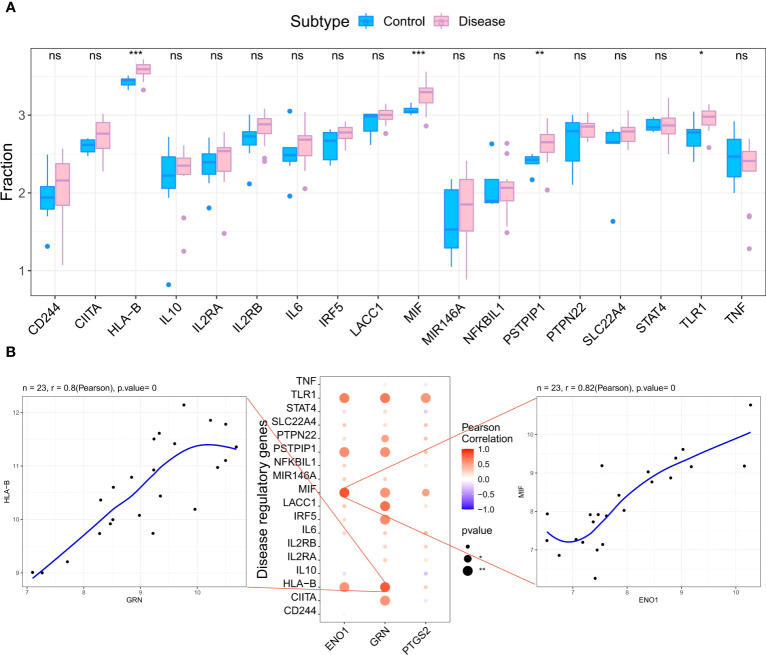
Analysis of the relationship between hub genes and the disease genes. **(A)** Differential analysis of disease regulatory genes. The regulatory genes of HLA-B, MIF, PSTPIP and TLR1 were significantly upregulated in RA compared to the normal control groups. (*p < 0.05 was considered significantly different **p < 0.01, ***p < 0.001, ns, no significance). **(B)** The correlation analysis of the hub genes and the differential regulatory genes. The first plot shows that GRN was significantly positively correlated with HLA-B. The second pot visualizes the Pearson correlation between hub genes and differential regulatory genes. The third plot shows PTGS2 was significantly positively correlated with MIF. The Pearson coefficients and p-values are shown at the top of the plots (p < 0.01 indicates significant correlation).

We retrieved 4881 RA-related pathogenic genes from the GeneCards database (https://www.genecards.org/ ), and analyzed the expression levels of the top 20 genes and found that the expression of HLA-B, MIF, PSTPIP, TLR1 differed in the two groups (**Figure 6A**). We then carried out a correlation analysis of key genes and genes regulating RA. and found that the expression levels of hub genes were significantly correlated with several RA-related genes. Specifically, ENO1correlated significantly and positively with MIF (Pearson r=0.82) and GRN correlated significantly and positively with HLA-B (Pearson r=0.8) (**Figure 6B**).

### 4.5 Identification of the pathogenic regions of hub genes by GWAS analysis

**Figure 7:**
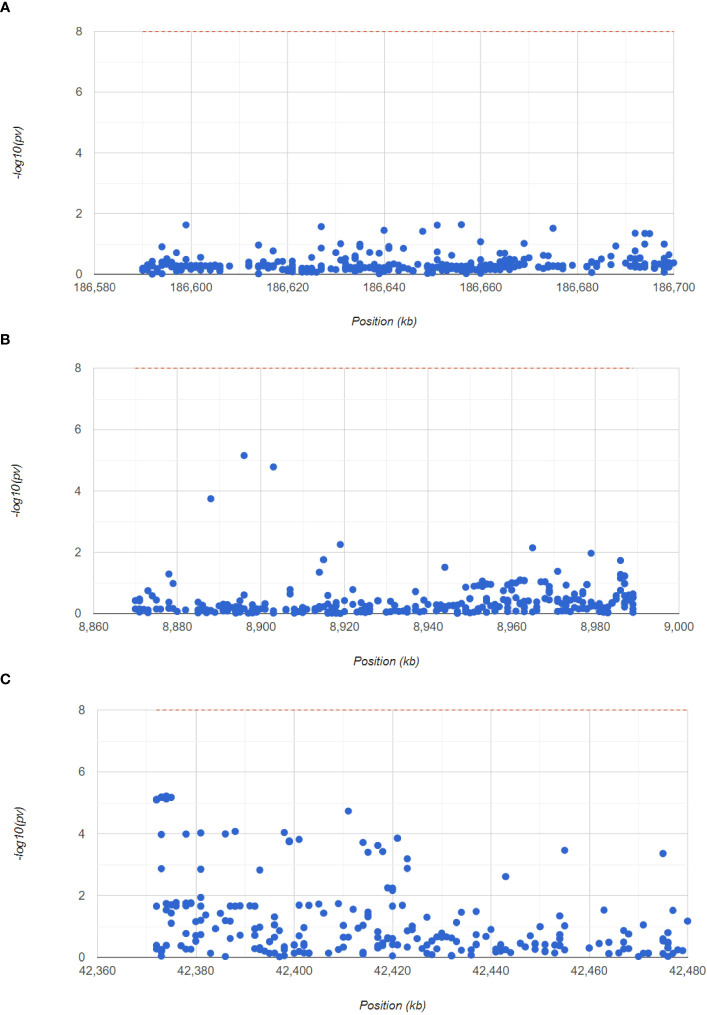
GWAS analysis to identify the pathogenic regions of the hub genes. The Manhattan plot shows the SNP pathogenic regions corresponding to PTGS2, ENO1 and GRN.

Next, to confirm the pathogenic regions of three hub genes in RA, we analyzed the RA GWAS data. We also showed the pathogenic regions of the SNPs corresponding to PTGS2, ENO1, and GRN, with PTGS2 and ENO1 located in chromosome 1 pathogenic region, and GRN in chromosome 17 pathogenic region (**Figure 7**). **Table S3** (GWAS data.xls) shows the significant SNP loci corresponding to the 3 genes.

### 4.6 Identification of the potential mechanisms of the hub genes by GSEA

**Figure 8:**
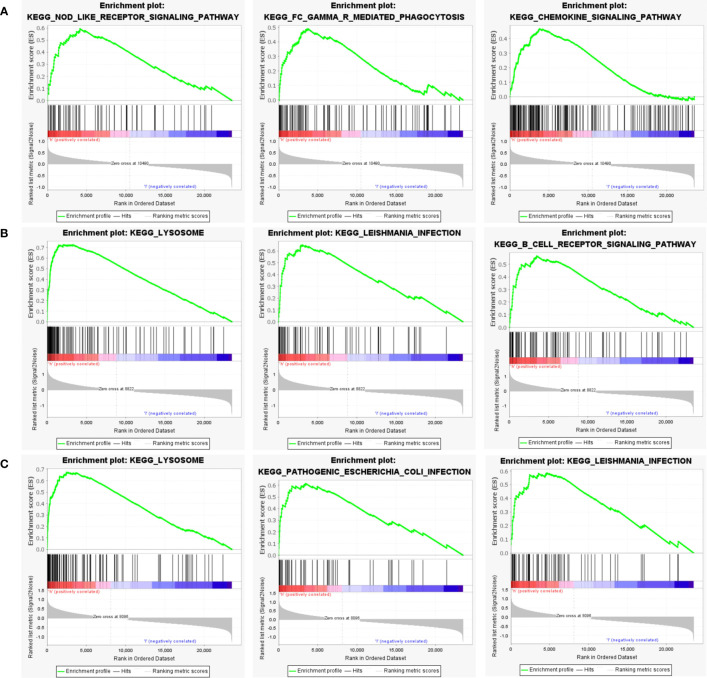
GSEA analysis of hub genes. **(A)** The top 3 most enriched pathways about function enrichment of PTGS2 gene: Nod-like receptor signaling pathway, gene set enriched in FcγR mediated phagocytosis, gene set enriched in the Chemokine signaling pathway. **(B)** The top 3 most enriched pathways about function enrichment of GRN gene: Lysosome, Leishmania infection, B cell receptor signaling pathway. **(C)** The top 3 most enriched pathways about function enrichment of ENO1 gene: Lysosome, Pathogenic Escherichia coli infection, Leishmania infection. Screening criteria for significant gene sets included adj. p-value < 0.05 and FDR < 0.5. NES, normalized enrichment score.

Next, to explore the underlying mechanisms of how these key genes affect disease development, we investigated the specific pathways associated with the three key genes. GSEA results revealed that the pathways enriched by the highly expressed PTGS2 gene are Nod-like receptor signaling pathway, FcγR mediated phagocytosis, Chemokine signaling pathway and other pathways (**Figure 8A**); high expression of GRN gene was enriched in Lysosome, Leishmania infection, B cell receptor signaling pathway and other pathways (**Figure 8B**); high expression of ENO1 gene was enriched in Lysosome, Pathogenic Escherichia coli infection, Leishmania infection and other pathways (**Figure 8C**).

### 4.7 Enrichment analysis for transcription factors of hub genes and construction of miRNA-mRNA regulatory network

**Figure 9:**
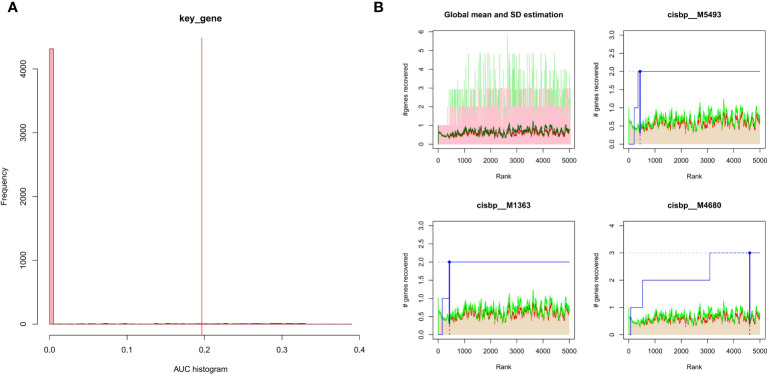
Enrichment analysis for transcription factors of hub genes **(A)** Histogram of the AUC. By calculating the AUC, the over-representation of each motif for hub genes was assessed. Red vertical line indicates the degree of significance, whereby motifs with AUC higher than the significance level are considered significant motifs. **(B)** The recovery curve for the three most significant motifs. Red line represents the global mean of recovered motif curve, green line represents the mean ± standard deviation. Motifs that were larger than the mean ± standard deviation were considered statistically significant. Blue line represents the current motif’s recovered curve. The motif cisbp_M5493 was significantly enriched in hub genes (ENO1 and PTGS2).

**Figure 10:**
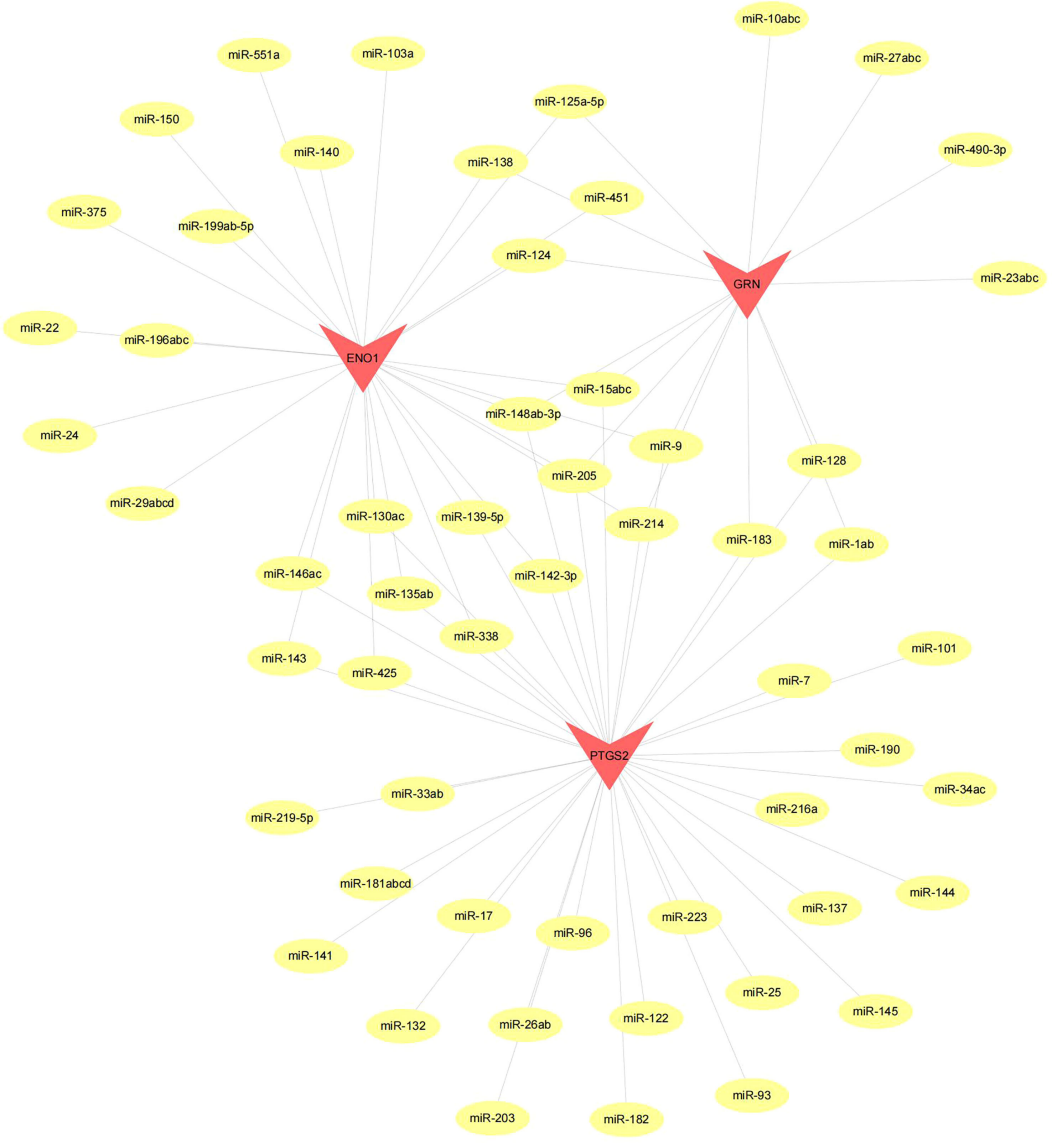
Network of miRNA–mRNA regulatory. The red triangle indicates the hub genes and yellow circle represent the candidate miRNA that targeted hub genes.

The 3 hub genes used for the analysis were found to be under the control of multiple TFs, as well as other common mechanisms. Therefore, an enrichment analysis was conducted for these TFs using cumulative recovery curves (**Figure 9**), motif TF annotation and key gene screening. The result revealed that the motif cisbp_M5493 had the highest normalized enrichment score (NES)of 6.17. Genes of ENO1 and PTGS2 were enriched in this motif. We showed the enriched motifs and the corresponding TFs (**Supplement Figure 1**). We also reverse predicted the 3 key genes using the miRcode database and obtained 56 miRNAs, a total of 80 mRNA-miRNA relationship pairs, and visualized them using Cytoscape (**Figure 10**). In addition, we utilized the miRcode database to reverse-predicet the three key genes and identify 56 miRNAs. We then visualized the resulting 80 mRNA-miRNA relationship pairs using Cytoscape, as shown in **Figure 10**.

### 4.8 Expression of hub genes in scRNA-seq

**Figure 11:**
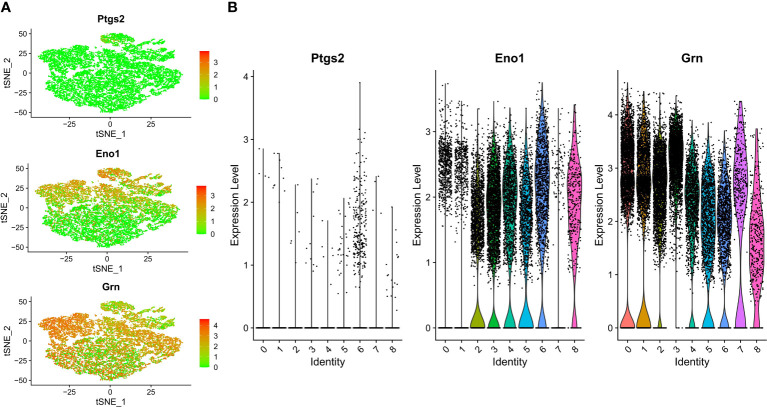
Single-cell analysis revealed the expression of PTGS2, ENO1 and GRN. **(A)** Dot plot of hub genes for each cell type. **(B)** Violin plots showing the expression level of hub genes in nine cell type.

Cells were clustered using the tSNE algorithm, and the expression of three key genes, prostaglandin-endoperoxide synthase 2 (PTGS2), enolase 1(ENO1), and granulin (GRN), in the nine cell clusters is shown in **Figure 11**.

### 4.9 Potential drugs prediction by connectivity map

**Figure 12:**
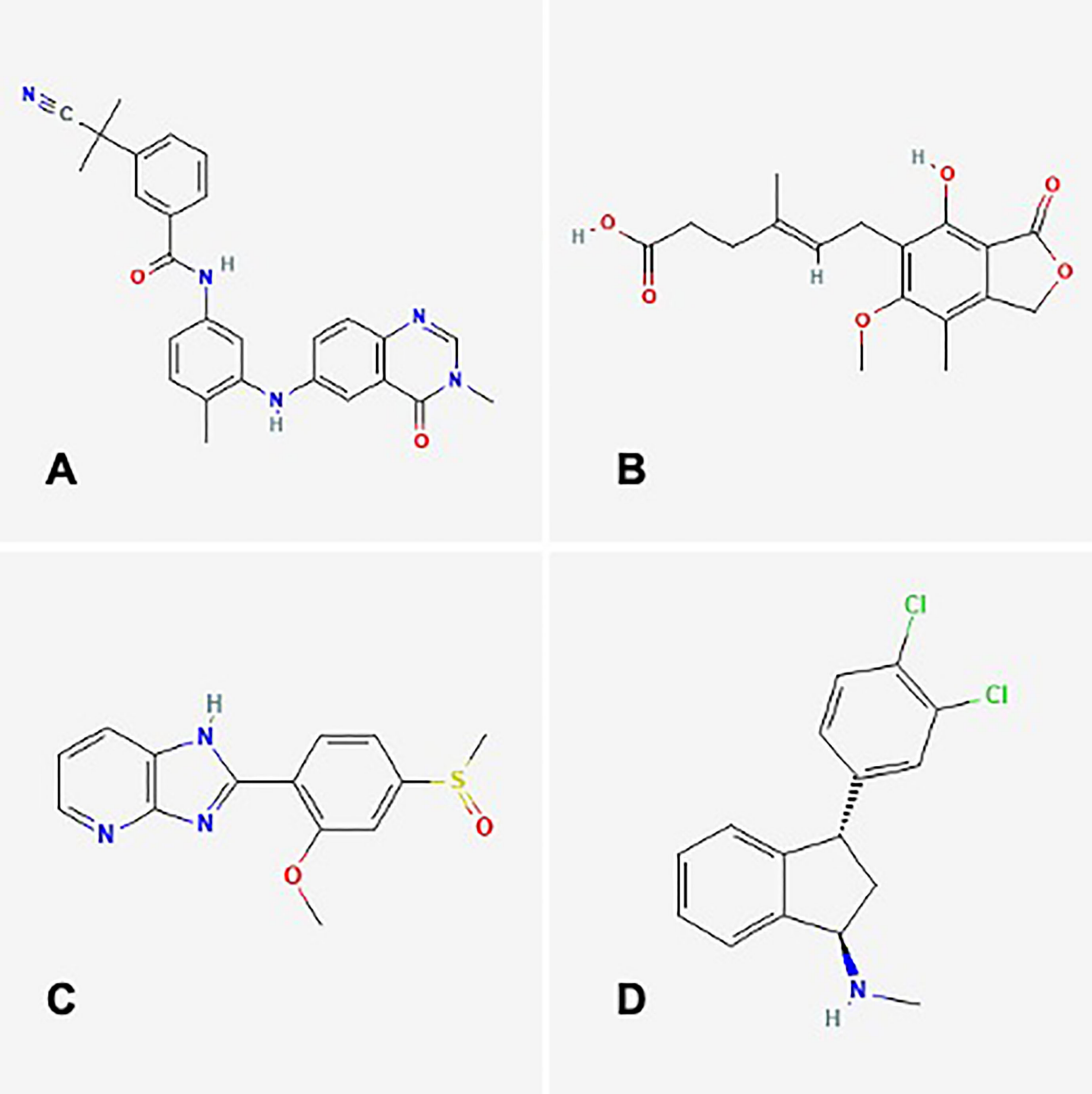
Drug predication using cMAP. **(A)** CID_11676786 **(B)** Mycophenolic acid **(C)** Sulmazole **(D)** Indatraline.

We utilized the Connectivity Map database to predict potential drugs for the disease. Our analysis revealed that the expression profiles of CID_11676786 (AZ628), mycophenolic acid, sulmazole, and indatraline drug perturbations had the most significant negative correlations with disease perturbation expression profiles. These findings suggest that these drugs could alleviate or even reverse the disease state (**Figure 12**).

### 4.10 Validation of the hub genes through western blotting(WB) and immunohistochemistry (IHC)

**Figure 13:**
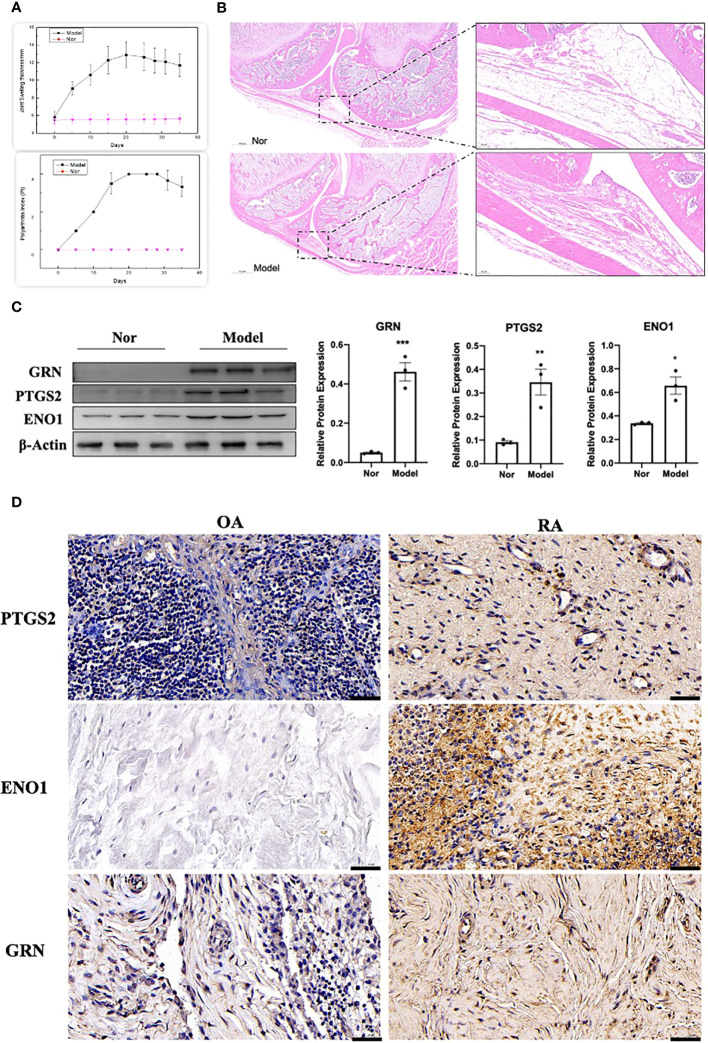
Validation of the hub genes in experimental samples. **(A)** Representative tracing of the PI and the joint swelling thickness in rat after CFA or Nor treatment in the two groups(n=12). **(B)** Representative images of H&E staining of joint sections from AIA rat or Nor rat. **(C)** The expression of GRN, PTGS2 and ENO1. n = 3. *p < 0.05, **p < 0.01, ***p < 0.001. **(D)** IHC staining for PTGS2, ENO1, and GRN on synovium from samples of OA and RA patients (n = 3).

The adjuvant-induced arthritis (AIA) rat model is a well-established and widely used method for establishing preclinical model of RA. After 25 days of exposure to Complete Freund’s adjuvant (CFA), the establishment of the model was evaluated by calculating the polyarthritis index (PI), joint swelling thickness and joint histopathology. The results demonstrated that model rats exhibited a significant increase in PI and joint swelling thickness, compared to control rats (**Figure 13A**). Furthermore, joint histopathological examination revealed that the synovial tissue in the model group was markedly hyperplastic, with thickened cells in the synovial lining layer, obvious inflammatory cell infiltration, and pannus formation (**Figure 13B**). We isolated and cultured primary FLS from rats and detected their protein expression levels using WB. As shown in **Figure 13C**, the expression of GRN, ENO1 and PTGS2 were significantly upregulated in the model group, which was consistent with the results in the RA patient datasets. To validate our findings in human samples, we further investigated the expression of GRN, ENO1 and PTGS2 through IHC in patient samples and found that these genes were dramatically elevated in RA compared to OA as a control (**Figure 13D**).

### 4.11 Potential mechanism of ENO1 in the regulation of ferroptosis

**Figure 14:**
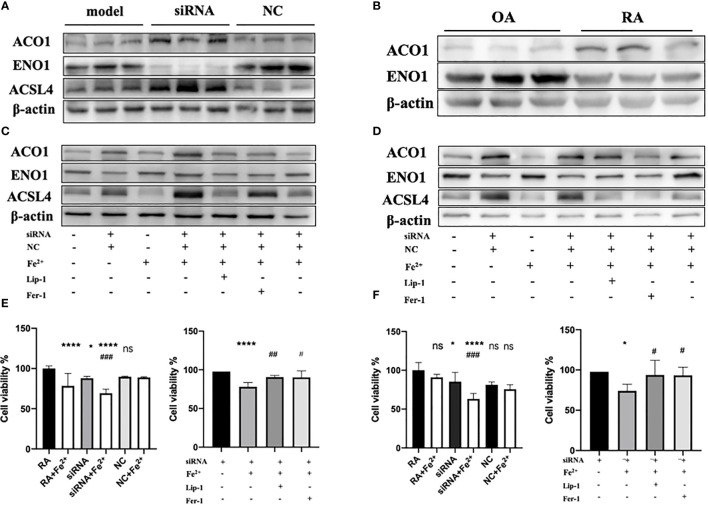
**(A)**The expression level of ENO1, ACO1 and ACSL4 in primary FLS of rat model with knockdown of ENO1-siRNA (n=3). **(B)** The expression level of ENO1 and ACO1 in primary FLS of RA patients(n=3). **(C, D)** The expression level of ENO1, ACO1 and ACSL4 in primary FLS of rat model and RA patients with knockdown of ENO1-siRNA in the presence of ferrous iron (200 μmol/L), Lip-1 (1 μmol/), Fer-1 (1 μmol/) (n=3). **(E)** The assessment of cell viability by CCK-8 assay in primary FLS of rat models with knockdown of ENO1-SiRNA in the presence of ferrous iron (200 μmol/L), Lip-1 (1 μmol/), Fer-1 (1 μmol/). Data are means ± SD. *P < 0.05, ****P < 0.0001 and *ns (no significance)* versus RA group; *^###^P < 0.001* versus siRNA group. ****P < 0.0001 versus siRNA group; #P < 0.05 and *^##^P < 0.01* versus siRNA+Fe2+group. **(F)** The assessment of cell viability by CCK-8 assay in primary FLS of RA patients with knockdown of ENO1-SiRNA in the presence of ferrous iron (200 μmol/L), Lip-1 (1 μmol/), Fer-1 (1 μmol/). Data are means ± SD. *P < 0.05, ****P < 0.0001 and *ns (no significance)* versus RA group; *###P < 0.001* versus siRNA group. *P < 0.05 versus siRNA group; *^#^P < 0.05* versus siRNA+Fe^2+^group.

**Figure 15:**
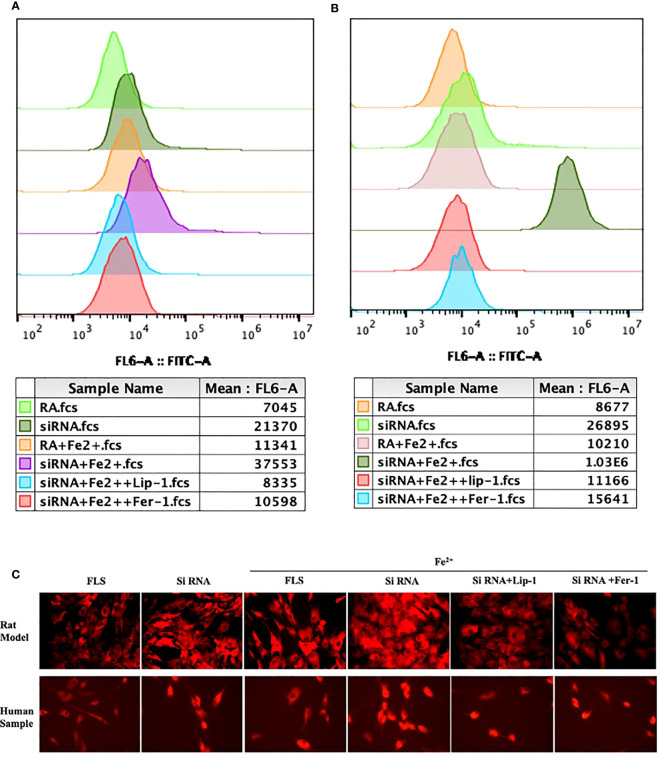
The assessment of lipid peroxidation by flow cytometry in primary FLS of rat model **(A)** and RA patients **(B)** with knockdown of ENO1-siRNA in the presence of ferrous iron (200 μmol/L), Lip-1 (1 μmol/), Fer-1 (1 μmol/). **(C)** The assessment of Intracellular ferrous ion by cellular ferrous ion detection fluorescent probes in primary FLS of rat model and RA patients with knockdown of ENO1-SiRNA in the presence of ferrous iron (200 μmol/L), Lip-1 (1 μmol/), Fer-1 (1 μmol/).

Since PTGS2 has been reported as a specific biomarker for ferroptosis (10), we focus on ENO1 to validate its potential function. ENO1, as an RNA-binding protein, can accelerate the messenger RNA decay of ACO1 in cancer cells, leading to repression of ferroptosis (15). ACO1 plays a critical role in the regulation of ferroptosis, which is involved in the activation of ferroptosis by ROS and the upregulation of genes that promote iron uptake and storage (16). We examined the expression of ENO1 and ACO1 in the synovium of RA patients, finding that ENO1 was upregulated while ACO1 was downregulated (**Figure 14B**). To further explore this pathway, we knocked down ENO1 in primary FLS of a rat model (**Figure 14A**), and found that the expression of ACO1 and the signature protein for ferroptosis, ACSL4, were increased. This effect was more pronounced in the presence of iron, and was reversed by treatment with ferrostatin-1(Fer-1) and Liproxstatin-1(Lip-1), both well-known ferroptosis inhibitors (**Figure 14C**). We also knocked down ENO1 in primary FLS of RA patients, leading to the upregulation of ACO1 and ACSL4. However, this effect was not observed to be more pronounced in the presence of iron. Treatment with Fer-1 and Lip-1 was still found to eliminate the increased ACSL4(**Figure 14D**). Moreover, consistent with previous reports that increased ACO1 led to excess accumulation of ROS and cell death in cancer cells (16, 17), knockdown of ENO1 led to increased cell death (**FigureS 14E, F**), enhanced lipid ROS generation (**Figures 15A, B**), and increased accumulation of intracellular ferrous ion (**Figure 15C**), particularly in the presence of iron. Treatment with Fer-1 and Lip-1 abolished ENO1-ACO1-mediated intracellular ferrous ion, ROS accumulation, and cell death (**Figures 15A–C**).

## 5 Discussion

In the progression of RA, increased iron levels in synovial tissue and fluid and increased chondrocyte ferroptosis have been observed in RA patients. Excessive synovial proliferation is a critical event in the pannus formation and cartilage damage. Ferroptosis has been considered to play a pivotal role in maintaining the balance of proliferation and death in the synovium.

Here, we identified 49 ferroptosis-related genes. Enrichment analysis revealed that ferroptosis-related genes were primarily involved in pathways including small molecule biosynthetic processes, metal ion response, and oxidoreductase activity. ENO1, GRN and PTGS2 were identified as hub genes of RA using the PPI network. ENO1 (α-enolase 1), an RNA-binding protein, is a critical glycolytic enzyme. It is known that ENO1 regulates malignant phenotype and cell proliferation through apoptosis (18, 19) and autophagy (20). Lee et al. reported that immune cells of RA patients express higher levels of ENO1 on their surface compared to the health, and that ENO1 triggers an enhanced pro-inflammatory response by interacting with ligands such as plasminogen and ApoB (21)Tong Zhang et al. found that ENO1 recruits CNOT6 to accelerate ACO1 mRNA degradation in cancer cells, resulting in inhibited mitoferrin-1 expression and subsequent suppression of ferroptosis (15). These findings suggest that ENO1 may be involved in RA progression by regulating ferroptosis,in addition to influencing the immune response. The GRN gene encodes the glycoprotein progranulin (PGRN) protein, which is expressed in a variety of cell types, including chondrocytes, endothelial cells, skeletal muscle cells and neurons (22–24). Full-length PGRN has been found to be anti-inflammatory as an antagonist of endogenous TNF-α by binding to TNFR (25, 26) However, it has been suggested that the cleaved granulin units may promote inflammation and counteract PGRN’s anti-inflammatory activity (27, 28). Increased risk of RA and joint destruction in PGRN^-/-^ mice model of collagen-induced arthritis compared to controls, and inflammatory arthritis can be revered by the administration of PGRN (25, 29, 30). Recently, Ting Chen et al. reported that PGRN upregulation attenuated ferroptosis by decreasing malondialdehyde and increasing Gpx4, Nrf2, and Slc7a11 expression and glutathione levels in microglia (31). Although the function of PGRN in the progression of RA is not fully understood, we can speculate that PGRN may influence RA progression by attenuating ferroptosis. The expression of PTGS2 (also known as COX2) is increased when ferroptosis occurs in cells. Co-treatment with Fer-1 inhibits the up-regulation of PTGS2, suggesting that PTGS2 is a functional biomarker of ferroptosis. It is therefore commonly used as an indicator of ferroptosis (32, 33). Zhou et al. have confirmed that inhibition of chondrocyte ferroptosis protects against articular cartilage damage and that PTGS2 is increased in articular cartilage (10). Activated synovial fibroblast is one of the dominant cell types in the hyperplastic tissue of RA. They are responsible for inflammation, matrix degradation, and angiogenesis through the production of proinflammatory cytokines, matrix-degrading enzymes and proangiogenic factors. Ferroptosis has been reported to be a contributor to its proliferation or death (8, 14). Wu et al. reported that the combination of TNF inhibitors and ferroptosis inducers may serve as a potential therapeutic strategy for RA therapy (8). This seems to be key implications for RA therapy, as it may be possible to eliminate large groups of fibroblasts in the hyperplastic rheumatoid synovium by triggering propagative death through eliminating resistance to ferroptosis. ENO1 and PGRN have been reported to suppress ferroptosis in cancer cells or neurons, we found for the first time that these two genes associated with ferroptosis are up-regulated in RA. Whether they are ideal targets for eliminating the resistance of rheumatoid synovium induced by ferroptosis deserves further investigation. To highlight the expression characteristics of hub genes and their possible regulatory mechanisms in the pathogenesis of RA, we performed a comprehensive bioinformatic analysis of the candidate ferroptosis hub genes.

We observed a significant variation in immune cells between RA and normal specimens. It is thought that ferroptosis could be involved in the regulation of the immune system by affecting immune cell numbers or inducing immune system recognition leading to inflammatory cascades or specific responses. Therefore, through CIBERSORT algorithm, we found that hub genes were found to be highly correlated with immune cells., This suggests that hub genes have a strong association with the degree of immune cell infiltrates, playing a key role in the immune microenvironment. We found that the expression of HLA-B, MIF, PSTPIP and TLR1 differed between the two groups from the GeneCards database and that these RA-related genes correlated significantly with the expression levels of hub genes, with ENO1 correlated significantly and positively with MIF and GRN correlated significantly and positively with HLA-B. This finding further supports the role of ferroptosis in RA pathogenesis. GWAS studies have confirmed the MHC region to be the most potent genetic risk factor, with more than 100 non-MHC RA risk loci identified (3). Our results confirmed that PTGS2 and ENO1 located in chromosome 1 pathogenic region and GRN located in chromosome 17 pathogenic region. Genome-wide researches have revealed strong genetic associations between the HLA regions and RA progression, indicating the importance of antigen recognition in RA pathogenesis (4). GSEA was used to further explore the underlying mechanism by which hub genes influence disease development. And the results revealed that hub genes were mainly enriched in the inflammation-associated signaling pathway, lysosome, phagocytosis and infection. A growing body of evidence suggests that ferroptosis requires the autophagy machinery for its performance (34). The lysosomal and phagocytic enrichment indicates that hub genes may contribute to ferroptosis through excessive autophagy and lysosome activity, consistent with previous research. Additionally, we found that the hub genes were regulated by common mechanisms such as multiple TFs. ENO1, PTGS2 were enriched in the motif of cisbp:M5493.

We construct the AIA rat model to validate the expression of the three hub genes. Our results revealed increased expression of these hub genes in the AIA rat model, which was also confirmed by analyzing synovium samples from RA patients. Remarkably, this study is the first to report high expression levels of ENO1 in RA. To gain further insight into the involvement of ENO1 in the regulation of ferroptosis in the development of RA, we examined the expression of ENO1 and ACO1 in the synovium of RA patients. Our results indicated that ENO1 was upregulated, while ACO1 was downregulated in the synovium of RA patients. Subsequently, we performed knockdown experiments on ENO1 in primary FLS derived from both AIA rat model and RA patients. We found that knockdown of ENO1 led to an increase in the expression of ACO1 and ACSL4. Additionally, this effect was more pronounced in the presence of iron, and was reversed by treatment with Fer-1 and Lip-1. Interestingly, the differing sensitivity of primary FLS from rat and human origin to ferrous ion concentrations may explain the differences in this effect on ACO1 and ACSL4 expression, which needs to be further verified in experiments with different ferrous ion concentrations. Moreover, we observed that knockdown of ENO1 led to enhanced lipid ROS generation, increased accumulation of intracellular ferrous ion, and increased cell death, especially in the presence of iron. Treatment with Fer-1 and Lip-1 successfully abolished ENO1-ACO1-mediated intracellular ferrous ion, ROS accumulation, and cell death, suggesting that the ENO1-ACO1 axis may play a significant role in the regulation of ferroptosis in the development of RA. Further investigation is warranted to explore the underlying mechanisms and the therapeutic potential of targeting this pathway for the treatment of RA.

In summary, our study identified ENO1, GRN and PTGS2 as ferroptosis-related genes and provided insights into the potential molecular mechanisms regulating synovial ferroptosis and the immune microenvironment. Targeting these genes and ferroptosis resistance to reduce synovial fibroblast numbers may be an effective and promising therapeutic strategy with RA. There are some limitations to this study. First, the study used microarray analysis and all the results were based on the expression levels of genes. Differences at the genetic level of biomarkers do not necessarily reflect differences in protein-level function and mechanism. Second, the samples for analysis and validation were relatively small, which may affect the accuracy of the analysis. Furthermore, this study provides preliminary evidence of a correlation between ferroptosis and RA pathogenesis, experiments need to be designed to further elucidate their mechanisms of action, and further *in vivo* data and clinical trials are warranted to investigate the role of ferroptosis in RA progression. It is worth mentioning that ferroptosis has been reported to be associated with multiple diseases, including autoimmune diseases. Growing evidence suggests that early ferroptosis is accompanied by immunogenic cell death (ICD). Ferroptosis occurs in synovial tissue and may further induce inflammation. In terms of mechanism, ruptured ferroptosis cells can release damage associated molecular patterns (DAMPs) such as DNA and HMGB1 or some other lipid molecules including phospholipid oxide, 4-HNE, 8-OHdG, PGE2, LTB4, LTC4, LTD4, which can induce innate immunity during tissue damage (35–41). Therefore, whether ferroptosis plays different roles in different stages of RA progression is also a critical issue that needs to be emphasized in future research. Specially, figuring out the immunogenicity and identifying the precise *in vivo* biomarkers and physiological signals that serve to induce or inhibit ferroptosis in the context of autoimmune disease will be of fundamental importance in determining the physiological function and therapeutic potential of ferroptosis. It is speculated that more direct evidence implicating ferroptosis in RA pathogenesis may emerge in the future.

## Data availability statement

The datasets presented in this study can be found in online repositories. The names of the repository/repositories and accession number(s) can be found in the article/**Supplementary Material**.

## Ethics statement

The animal study was reviewed and approved by Medical Ethics Committee of Peking University First Hospital (protocol code: J2022108, date of approval: 17 November 2022).

## Author contributions

XH and CZ developed the ideas and drafted the manuscript. YC and XP obtained funding and supported the experiment design. JZ and MG were responsible for partial data analysis and experiments, YG and BD acquired the data and revised the manuscript. All authors contributed to the article and approved the submitted version.
