# Supplementary material for: Identification of potential ferroptosis-associated biomarkers in rheumatoid arthritis
Source: Front Immunol. 2023 Jul 10;14:1197275. doi: 10.3389/fimmu.2023.1197275 (PMC10364059; doi:10.3389/fimmu.2023.1197275)
Supplement: Supplementary file 1 [file Table_1.docx]

**Table S1** Demographic, clinical, and serological characteristics of specimens from OA and RA patients

|  | OA | RA |
| --- | --- | --- |
| Number, n | 3 | 3 |
| Male/Female, n | 1/2 | 1/2 |
| Age (years) | 66 [58,74] | 72 [63,82] |
| BMI (kg/m^2^) | 19.7 [17.1,21.1] | 18 [15.4,21.7] |
| CRP (mg/L) | 6.83 [1.63,15.7] | 9.19 [1.48,13.6] |
| ESR (mm/h) | 34.67 [9,69] | 45.33 [11,73] |
| RF (U/ml) | ND | 1082.67 [645, 1810] |
| Anti-CCP (RU/ml) | ND | 690 [170, 1110] |
| Duration of disease (years) | 13 [9,19] | 16.67 [12,21] |

Abbreviations: RA, rheumatoid arthritis; OA, osteoarthritis; BMI, Body Mass Index; ND, not determined; CRP, C-reactive protein; ESR, erythrocyte sedimentation rate; RF, rheumatoid factor; anti-CCP, anti-cyclic citrullinated peptide. Values for non-normally distributed measurements were expressed as median (minimum, maximum).
