# Supplementary material for: Identification of potential ferroptosis-associated biomarkers in rheumatoid arthritis
Source: Front Immunol. 2023 Jul 10;14:1197275. doi: 10.3389/fimmu.2023.1197275 (PMC10364059; doi:10.3389/fimmu.2023.1197275)
Supplement: Supplementary file 2 [file Table_2.docx]

**Table S2. Primer sequences of siRNAs and negative control**

| Species | Genes | Primer sequences |
| --- | --- | --- |
| Rat | ENO1-siRNA | GCUCAAAGUGAACCAGAUUTT AAUCUGGUUCACUUUGAGCTT |
| Human | ENO1-siRNA | CCAACAUCCUGGAGAAUAATT UUAUUCUCCAGGAUGUUGGTT |
|  | Negative control | UUCUCCGAACGUGUCACGUTT ACGUGACACGUUCGGAGAATT |

Abbreviations: ENO1, enolase 1; siRNA, small interfering RNA.
